# Understanding the Effects of Crosslinking and Reinforcement Agents on the Performance and Durability of Biopolymer Films for Cultural Heritage Protection

**DOI:** 10.3390/molecules26113468

**Published:** 2021-06-07

**Authors:** Giulia Infurna, Giuseppe Cavallaro, Giuseppe Lazzara, Stefana Milioto, Nadka Tzankova Dintcheva

**Affiliations:** 1 Department of Engineering, University of Palermo, Viale delle Scienze, Ed. 6, 90128 Palermo, Italy; giulia.infurna@unipa.it; 2 National Interuniversity Consortium of Materials Science and Technology (INSTM), Research Unit of Palermo, Via G. Giusti, 9, 50121 Florence, Italy; giuseppe.cavallaro@unipa.it (G.C.); giuseppe.lazzara@unipa.it (G.L.); stefana.milioto@unipa.it (S.M.); 3 Department of Physics and Chemistry, University of Palermo, Viale delle Scienze, Ed. 17, 90128 Palermo, Italy

**Keywords:** chitosan, pectin, citric acid, halloysite nanotubes, mechanical behaviors, optical properties, durability

## Abstract

In the last two decades, the naturally occurring polysaccharides, such as chitosan and pectin, have gained great attention having potential applications in different sectors, from biomedical to new generation packaging. Currently, the chitosan and pectic have been proposed as suitable materials also for the formulation of films and coatings for cultural heritage protection, as well as packaging films. Therefore, the formulation of biopolymer films, considering only naturally occurring polymers and additives, is a current challenging trend. This work reports on the formulation of chitosan (CS), pectin (PC), and chitosan:pectin (CS:PC) films, also containing natural crosslinking and reinforcement agents, such as citric acid (CA) and halloysite nanotubes (HNT), through the solvent casting technique. The produced films are characterized through water contact angle measurements, infrared and UV–visible spectroscopy and tensile test, while the durability of the CS:PC films is evaluated subjecting the film to accelerated UVB exposure and monitoring the photo-oxidation degradation in time though infrared spectroscopy. All obtained results suggest that both crosslinking and reinforcement agents have beneficial effects on the wettability, rigidity, and photo-oxidation resistance of biopolymer films. Therefore, these biopolymer films, also containing naturally occurring additives, have good properties and performance and they are suitable as coverage films for cultural heritage protection.

## 1. Introduction

The circular economy, with a framework of policies that make sustainable products, services, business models, and norms, is a well-delineated transition by the European Commission and EU Governances. However, the shifting towards more sustainable materials and production processes needs to be performed as soon as possible [1].

Therefore, the introduction of fully bio-based materials, although not fully biodegradable, to produce goods and services, is widely requested by a large part of public because of benefits for the environment and human health [2,3]. 

Naturally occurring polymers, such as polysaccharides, polyhydroxyalkanoates, polylactic acids, starches, proteins, and lipids, have applications mainly in the biomedical and pharmaceutical sectors, and food packaging sectors, and their properties and performance depend on the chemical nature and molecular weight of the monomers, polymerization degree, and production technologies. Among the biopolymers, the polysaccharides are the most important and popular class of polymers coming from natural sources, such as animals, seafoods, and vegetables; this is also due to their biocompatibility, biodegradability, and non-toxicity [4,5]. Among the polysaccharides, chitosan and pectin have attracted much attention because of their abundance in nature, relative low cost, and low environmental impact [6].

Chitosan is a copolymer of glucosamine and *N*-acetylglucosamine units, and it is derived by deacetylation in the presence of sodium hydroxide of chitin, which is the second most abundant polysaccharide in the world after cellulose, and large present in the exoskeleton of crustaceans, in fungal cell walls, and other biological materials [7]. The chitosan grade of deacetylation, molecular mass [8,9], and presence of plasticizers [7,10,11] are the determinants for its properties and performance, and obviously are determinant for chitosan applications, such as protective films, edible coatings for foods, and transparent films for cultural heritage protections because of its selective permeability to CO_2_ and O_2_ [7,12]. Chitosan is a cationic polysaccharide, as known, its glucopyranose rings coordinate positive charged groups, and it shows a tendency to interact with water molecules and active ingredients [13]. The main chitosan drawback is related to its ability to adsorb water molecules and to swell, and for this reason, usually, to improve the chitosan resistance to water molecules, a crosslinking through treatment by acids is carried out [14]. Currently, the use of citric acid as crosslinking agent for chitosan is proposed by Priyadarshi et al. [15], and the crosslinking mechanism is explained through the formation of covalent intermolecular di-ester linkage between carboxyl groups of citric acid and the chitosan hydroxyl groups.

Pectin is a typical liner heteropolysaccharides, formed by d-galacturonic acid linked by α-1,4 glycosidic bonds [16,17]. Pectin is widely considered as a good candidate for coatings for fruits and foods because of its biodegradability, non-toxicity, and edibility. As known, the pectin is an anionic polysaccharide, because of the presence of numerous negatively charged carboxylic groups in its chemical structure, and for this reason, it shows the tendency to interact with metal cations and active ingredients. According to literature, pectin may form a three-dimensional network structure by adding bivalent cations such as calcium ions, and the calcium ions may be packed in the interstices of the twisted pectin chains, which may improve the film performance [18].

Our previously published paper [19] was focused on the preparation of bio-nanocomposite films based on a chitosan and pectin blend, the investigation of theoretical solubility of pectin into chitosan, and the definition of the effective ratio between the two constituents. Based on the theoretical calculation using Hoy’s method, pectin is very soluble into chitosan. Interestingly, the effective ratio between the two constituents is 1:1 for the film formulation through solvent casting, based on the z-potential measurements, considering the different charges of both polysaccharides, and the resultant handling of the final film. Further, to introduce naturally occurring stabilizers, aiming to improve the stability of the bio-nanocomposite films, the halloysite clay were considered as carrier for vanillic acid and quercetin molecules [19,20]. Therefore, by adding natural compounds, such as halloysite clay and antioxidants, some performance and properties of polysaccharide films [21,22,23] can be improved and makeable for numerous challenging applications [24,25,26]. HNT is abundant in nature, relative low cost, and in the last few years it was proposed as a biocompatible smart material that can replace carbon nanotubes [27,28,29]. In fact, the interesting geometrical configuration of halloysite nanotubes, a rolled-up kaoline sheet with a positive internal surface charge, and a negative external surface charge [30], make HNTs suitable not only as a filler for biopolymer but also as a carrier in fields like drug delivery, tissue engineering, oil recovery, and eco-compatible packaging [31,32].

In this work, the effects of halloysite clay and citric acid, as efficient reinforcement and crosslinking agents, on the performance and durability of chitosan (CS), pectin (PC), and chitosan/pectin (CS:PC) blend, are investigated. Based on our previous experience, the CS:PC blend has been produced at 50/50 wt/wt%. The halloysite clay (HNT) and citric acid (CA) are added at 20 wt% and at 3 wt%, respectively, to the single CS and PC and to the CS:PC blend, and the films are prepared by solvent casting technique. The biopolymer films containing CA and HNT have improved wettability, rigidity, and photo-oxidation durability. The HNT slightly decreased the biopolymer film transparency, while the CA did not have any negative effect on the transparency, and both HNT and CA additives could be considered suitable additives for the formulation of films for cultural heritage protection. 

## 2. Materials and Methods

### 2.1. Materials

The materials used in this work are: Chitosan (CS), low viscosity, deacetylation degree = 75−85% and average molecular weight = 120 kg mol^−1^; Pectin (PC) from Citrus, Poly-d-galacturonic acid methyl ester with degree of methyl esterification 24%, Mw = 30−100 kg mol^−1^; Halloysite nanoclay (HNT), [Al_2_Si_2_O_5_(OH)_4_]x2H_2_O has Formula Weight = 294.19 g mol^−1^; Pore Volume = 1.26–1.34 mL/gm; pH = 4.5–7.0; Diameter = 30–70 nm; Length = 1–3 microns; Citric acid (CA), formula C_6_H_8_O_7_, white crystals, molecular weight = 192.12 g mol^−1^; all used materials were purchased by Sigma-Aldrich and used as received. 

### 2.2. Preparation of Bio-based Films

Aqueous solutions of each biopolymer at 2%wt were prepared following the procedure detailed elsewhere and equilibrated overnight [33,34]. The CS:PC blends, without and with HNT or CA, were prepared at 50/50 wt/wt%.

The HNT and CA were added to the single CS or PC or to the CS:PC blend solutions at 20 wt% and 3 wt%, respectively, and were kept under stirring overnight. The well dispersed aqueous mixture was poured into glass Petri dishes (15 g) under vacuum at room temperature to evaporate water until the weight was constant and to obtain biopolymer films with a thickness ranging from 80 to 100 μm.

### 2.3. Characterization

#### 2.3.1. FT-IR Analysis

A Fourier Transform Infrared Spectrometer (Spectrum One, Perkin Elmer, Shelton, CT, USA) was used to record IR spectra using 16 scans at a resolution of 1 cm^−1^. Measurements were obtained from the average of triplicate samples with a calculated maximum experimental error (relative standard deviation) of around 5%. The deconvolution of FTIR peaks was performed using the scientific software OriginPro 8.5.

#### 2.3.2. UV–visible Analysis

UV–visible Spectrometer, (Specord^®^250 Plus, Analytikjena, Torre Boldone, BG, Italy), was used to record UV–vis spectra performing 8 scans between 200 and 1100 nm at a resolution of 1 nm. The transparency of the films has been evaluated through calculation of the values of linear attenuation coefficient using the formula:

K = A_750nm_/(2.3 × S) (1)

where A is the absorbance values at 750 nm and S is the sample thickness.

#### 2.3.3. Contact Angle Measurements

The water contact angle was measured at room temperature by means of First Ten Angstrom (USA) FTA1000C system (Data Physics Instruments, Filderstadt, Germany), with demineralized water. The films were fixed on top of a plane solid support and kept flat during water deposition and acquisition. The sessile drop method was used with a droplet volume of 6 µL.

#### 2.3.4. Mechanical Characterization

Tensile tests were carried out using a Universal Testing Machine (Instron model 3365, Bucks, UK), following ASTM D882 method, on rectangular samples cut by films prepared by compression molding and solvent casting. The tests were performed using tensile speed at 1 mm/min. Young’s modulus (E), tensile strength (TS), and elongation at break (EB) were recorded, and the data reported represent the average values obtained by analyzing the results of eight tests per sample; the variability of mechanical tests was typically of order of ±5%.

#### 2.3.5. Photo-oxidation Exposure

Photo-oxidation of CS:PC films, about 80 μm thick, without and with HNT (at 20 wt%) and CA (at 3 wt%), was carried out using a Q-UV-Solar Eye weatherometer (from Q-LAB, Westlake, OH, USA) equipped with UVB lamps (340 nm). The weathering conditions were a continuous light exposure at T = 55 °C.

## 3. Results and Discussions

**Figure 1:**
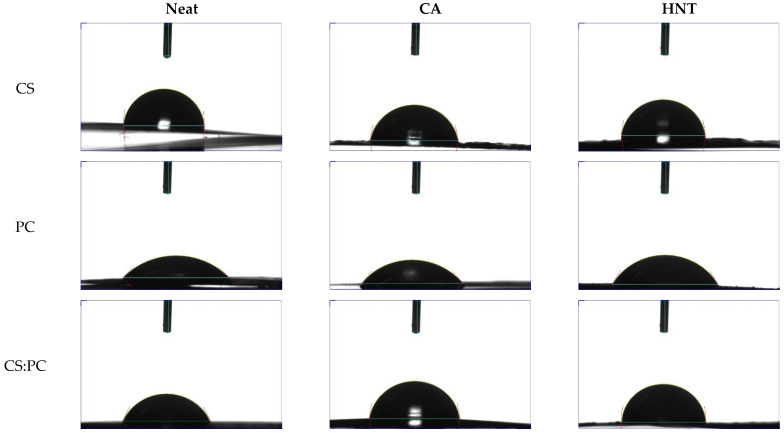
Frame of water contact angle (WCA) immediately after drop deposition on the biopolymer films surface.

To investigate the wettability of biopolymer films, also containing crosslinking and reinforcement agents, the water contact angle (WCA) measurements were performed, and in Figure 1 and Table 1, the frame of WCA after the drop deposition on the films surface and WCA values are reported. It is worth noting that neat CS shows the highest contact angle value in comparison to that of neat PC and CS:PC blend. The CS film shows the highest hydrophobicity, while the PC film is particularly hydrophilic. As expected, the CS:PC blend shows an average WCA value between the WCA values of neat CS and PC, suggesting that the presence of PC has a clear negative effect on the film wettability. Interestingly, the CA presence has a very positive effect on the PC wettability, improving the film hydrophobicity of both PC, and even more, the CS:PC blend. The hydrophobicity of CS slightly decreases due to the CA presence.

The presence of reinforcement agent, as HNT, exerts similar effect on the wettability of CS, PC, and CS:PC, to that induced by crosslinking agent, as CA, although the beneficial effect of HNT is even more pronounced that the effect of CA. Finally, the WCA of CS:PC/HNT and neat CS film are very similar, highlighting that the negative effect of PC on the wettability of CS:PC blend is overcome by the additives presence, even more by HNT.

**Figure 2:**
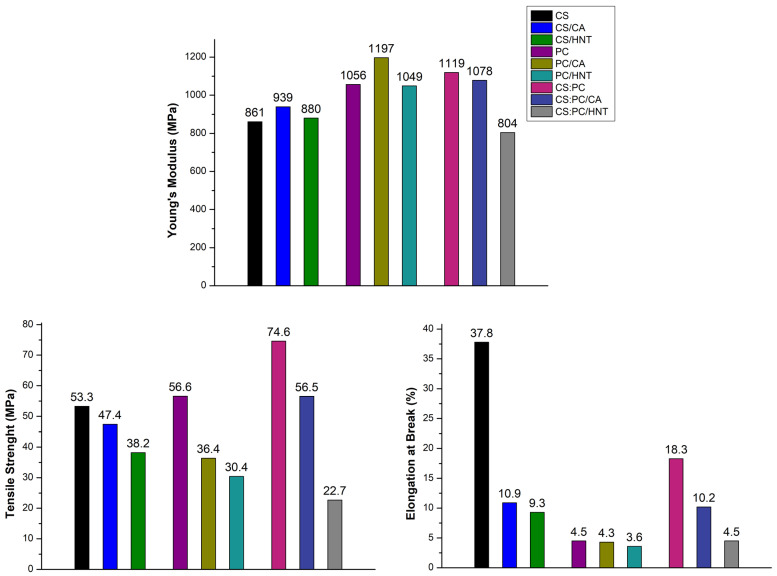
Main mechanical properties of all investigated samples.

To investigate the mechanical behavior, all investigated films are subjected to tensile test, and in Figure 2, obtained main mechanical properties, i.e., Youngs’ modulus (E), tensile strength (TS), and elongation at break (EB), are reported. It is worth noting that both E and TS values of the CS:PC blend are higher than the values of neat CS and PC, suggesting a good synergism between the two polysaccharides constituents in formulation of the blend film. Therefore, the EB value of the CS:PC blend is reduced two times in comparison to the EB value of neat CS, but this result is expected considering that the PC is totally brittle, i.e., its EB value is ca. 4.5%, see Figure 2. 

It seems that the CA presence in both neat CS and PC leads to a slight increase of E values, exerting a beneficial effect on the system rigidity, due to CA crosslinking action. The E values of both CS:PC and CS:PC/CA films are very similar even though the CA presence slightly decreases the rigidity (ca. 5% lower), but this decrease is almost imperceptible taking into account the application of the films as coverage for cultural heritage protection.

The HNT presence leads to a slight decrease of E values of CS, PC, and CS:PC, probably, because at this high amount (i.e., at 20 wt%), the dispersion is not favored due to HNT polar nature. Therefore, the enhancement of system heterogeneity, due to HNT presence, is even more pronounced for the CS:PC blend, and the latter is noticeable also for the TS and EB trend.

**Figure 3:**
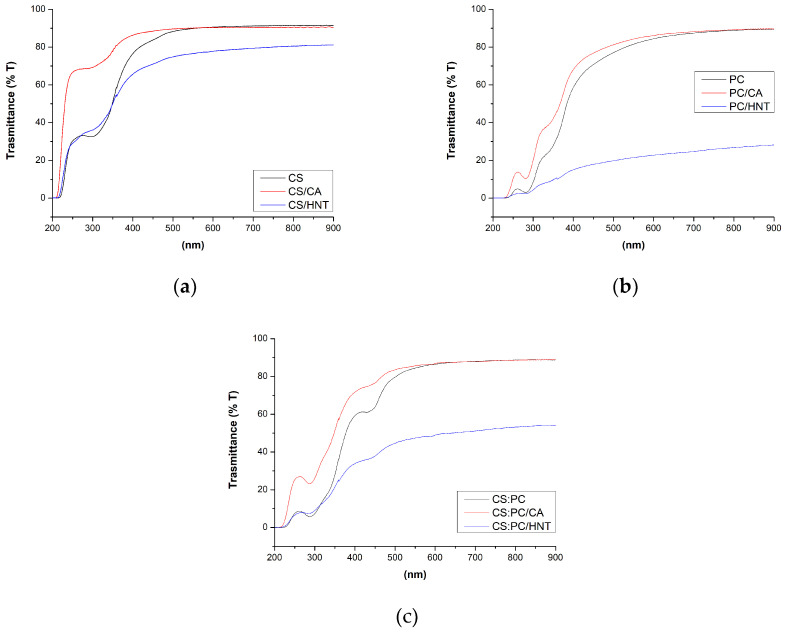
UV–visible spectra of biopolymer films containing citric acid (CA) or halloysite nanotubes (HNT) in which the matrix is (**a**) chitosan, CS, (**b**) pectin, PC, (**c**) chitosan:pectin blend, CS:PC.

In Figure 3, the UV–visible spectra of CS, PC, and CS:PC films, containing also CA and HNT, are plotted. Additionally, using Equation (1) that is reported in the experimental section, the values of linear attenuation coefficient (*K_750_*) of all investigated biopolymer films are calculated and reported in Table 2. It is clearly noticeable that the CA presence nearly does not modify the transparence in both the UV and visible ranges, highlighting a beneficial effect on the optical performance of the biopolymer films, due to the presence of the crosslinking agent. The latter suggests that the biopolymer films containing citric acid are transparent and can be considered good candidates as coverages for cultural heritage protection. 

It is worth noting that the HNT presence has a pronounced effect on the biopolymer film transparence. Unfortunately, the HNT at this very high amount, leads to a significant decrease of the transparence in the visible range, suggesting the possibility that these films could be employed where high transparence is not requested. It is noticeable that the HNT presence decreases the transparency of CS film, and even more of PC film. The observed transparency decrease for CS:PC (HNT blend film) is slightly higher than that of CS/HNT film, and absolutely lower than that of PC/HNT.

**Figure 4:**
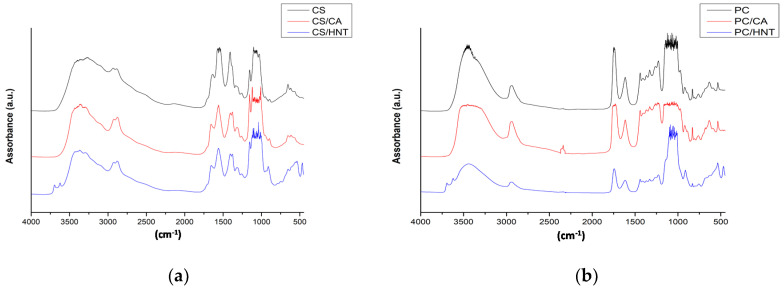

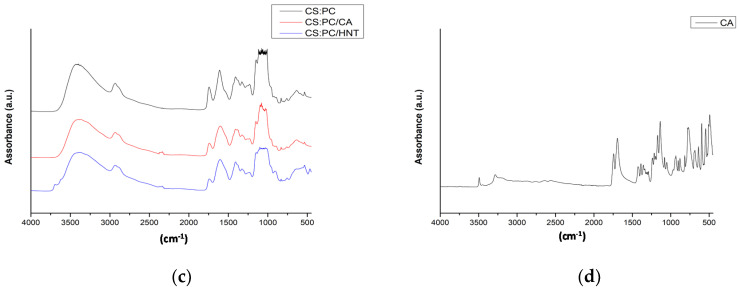
FT-IR spectra of biopolymer films containing citric acid (CA) or halloysite nanotubes (HNT) in different matrices: (**a**) chitosan, CS, (**b**) pectin, PC, (**c**) chitosan:pectin blend, CS:PC; (**d**) ATR-FTIR spectrum of neat CA.

In Figure 4a–c, the FTIR spectra of CS, PC, and CS:PC films, containing also CA and HNT, are plotted, and in Table 3, according literature, the assignment of main absorption peaks is reported. It is noticeable that the presence of CA and HNT, does not have a significant influence on the main peak positions. However, according to literature and as noticeable in Table 3, the different absorption bands in the FTIR spectra of CS and PC are very similar, because of the similarity of the groups in the structure of both polysaccharides. Indeed, the FTIR spectra of the CS:PC blend appears very similar to the spectra of neat CS and PC, and there is overlapping of numerous absorption bands with these of neat CS and PC.

Therefore, no additional band, due to the CA presence, is noticed in the IR spectra, see Figure 4d, because the CA intrinsic chemical groups are similar to that of both CS and PC structures. As noticeable in Figure 4d, where the ATR-FTIR spectrum of neat Ca is shown, the CA shows main absorption bands in the hydroxyl range at ca. 3560 and 3290 cm^−1^; in the carbonyl range at ca. 1721 and 1710 cn^−1^; and other bands at ca. 1208, 1105, 779, and 596 cm^−1^; and these bands are overlapped by the bands of both CS and PC intrinsic structures and the detection by FTIR of crosslinking occurrence is not easy. For this reason, the neat films (CS, PC, and CS:PC) and CA-containing films were immersed in double deionized water. Neat CS, PC, and CS:PC films are totally soluble into the water after 24 h, while the CS/CA and CA:PC/CA show a residual weight at ca. 12 wt% and ca. 8 wt%, respectively. For PC/CA film, after 24 h in water, no residual weight is detected, suggesting the occurrence of physical interactions, rather than chemical crosslinking reactions. 

It seems that, due to the presence of HNT at high concentration, the absorption of the main bands of both neat matrices CS and PC, and of the CS:PC blend, appear lower, and two small peaks at around 3700–3600 cm^−1^ are noticed. According to literature, in FTIR spectra of alumino-silicates, peaks appear at around 910 cm^−1^ and small shoulder at around 3700 cm^−1^, due to metal-oxide structures and coordinated hydroxyl groups, respectively. The absorption band at 910 cm^−1^ is partially overlapped by different small peaks in this range 950–890 cm^−1^ due to intrinsic structures of both polysaccharides (see the assignment of FTIR absorption bands in Table 3).

To evaluate the durability of CS:PC blend, containing also CA and HNT, thin films were subjected to accelerated UVB exposure. The photo-oxidative degradation in time of CS:PC, CS:PC/CA, and CS:PC/HNT films was monitored through FTIR analysis at regular intervals at about 8 h. According to literature, chitosan degradation mechanism occurs mainly by depolymerization, followed by deacetylation, oxidation, and interchain crosslinking [35], while pectin depolymerization proceeds mainly by backbone hydrolysis, β-elimination, and decarboxylation [36].

However, according to our previous work [19], the adding of HNT to biopolymer matrices leads to the formulation of suitable bionanofilms as coverage for cultural heritage protection, although the film transparence is slightly penalized. As known, the HNT protect efficiently the polymer matrices against ultraviolet exposure, because the alumino-silicates structure can absorb UV irradiation, and polymer-based systems containing HNT have improved photo-oxidation resistance [19,37,38,39]. Therefore, the HNT marked absorption ability, especially if the HNT are added at high concentrations, could induce the decrease of the transparence in the visible range, and this could be taken into account for some specific film applications, where high transparency in the visible range is required.

Therefore, the CA adding results in the formulation of crosslinked biobased films with improved oxidation resistance [40,41]. Taking into account our previous experience, in this work, the photo-oxidation behavior of the CS:PC blend, also in the presence of CA and HNT, is investigated monitoring the structural changes in time by infrared spectroscopy.

However, as discussed above, the CS:PC spectrum, also before UVB exposure, presents numerous multiple peaks, and considering the complexity of degradation phenomena of both neat CS and PC, the investigation of photo-oxidation resistance of CS:PC films through FTIR analysis is a hard matter.

**Figure 5:**
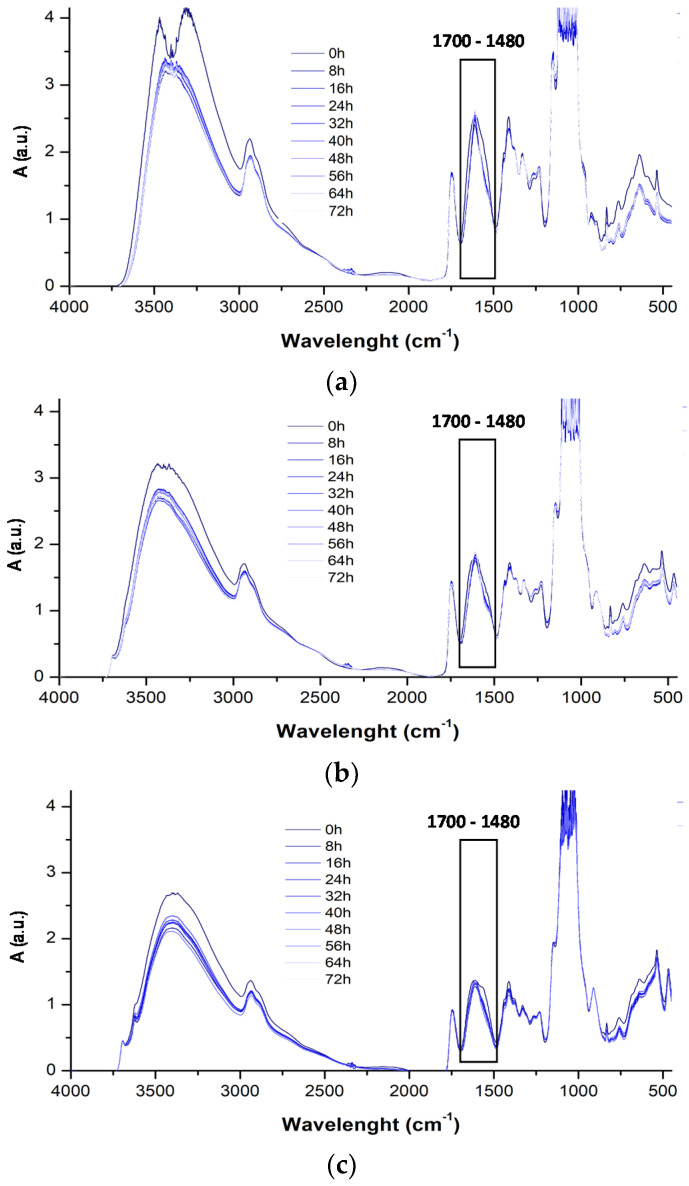
FTIR spectra at different exposure time of (**a**) CS:PC, (**b**) CS:PC/CA and (**c**) CS:PC/HNT films.

**Figure 6:**
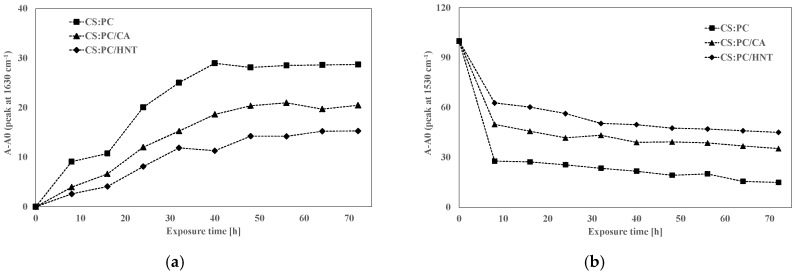
Variation of (**a**) >C=O (~1630 cm^−1^) and (**b**) NH bending (~1530 cm^−1^) for CS:PC, CS:PC/CA and CS:PC/HNT films.

The photo-oxidative degradation of CS:PC blend can be profitably followed monitoring the changes in the spectra in range 1700–1480 cm^−1^ in time, which contains two different peaks: first peak at around 1630 cm^−1^ due to the C=O presence of COO (ester) ion stretching of secondary amide group (amide I) for CS (~1626 cm^−1^) and -O(-)-C=O, for PC (~1634 cm^−1^), and expectedly, the peaks due to C=O stretching for both CS and PC appear a single peak and second peak at around 1530 cm^−1^ attributed to N-H bending, according to literature [19]. In Figure 5a–c, the FTIR spectra of CS:PC, CS:PC/CA and CS:PC/HNT, obtained at different exposure time, are plotted. Considering the complexity of the degradation phenomena and based on our previous experience, the complex bands in the range 1700–1480 cm^−1^ are deconvoluted, and additionally, in Figure 6a,b, the variation of >C=O (~1626 cm^−1^) and NH bending (~1533 cm^−1^) trends, respectively, for CS:PC, CS:PC/CA and CS:PC/HNT films are plotted. It is worth noting that both HNT and CA have protective actions towards the CS:PC blend, suggesting their beneficial effects on the films durability. Interestingly, based on the trends reported in Figure 6, the HNT can protect more efficiently, than the CA, the bio-based films against the UVB exposure. Therefore, the HNT protection efficiently, against UVB exposure, could be understand considering the marked absorption ability of the nanoclay inorganic nature, i.e., alumino-silicates structure, as detected by UV-visible analysis and above commented.

However, although the degradation mechanism of CS:PC is a very complex phenomena, there can be supposed that the simultaneous CS depolymerization and subsequent deacetylation and oxidation and PC depolymerization and subsequent decarboxylation,t that lead to >C=O accumulation, see Figure 6a. Therefore, the CA and HNT slow down the >C=O accumulation acting differently, specifically, the CA favors the formation of crosslinked structures, while the HNT absorb efficiently the UV irradiation. By the decrease of NH bending trends, see Figure 6b, it could be supposed a reaction occurrence between the -NH radicals and new oxygen-containing species, both coming from simultaneous occurrence of depolymerization and oxidations.

## 4. Conclusions

Chitosan, pectin, and chitosan-pectin films, also containing crosslinking and reinforcement agents, are successfully formulated, and their properties and performance are accurately investigated. It seems that the presence of both citric acid and halloysite nanotubes have a beneficial effect on the film performance, specifically, the CA does not modify the optical properties, while the HNT have a beneficial effect on the rigidity of the biopolymer films. In addition, the adding of both CA and HNT extend the CS:PC durability, specifically, the CA favors the formation of crosslinked structures, while the HNT absorb efficiently the UV irradiation.

To sum up, all formulated biopolymer films can be considered suitable coverages for cultural heritage protection. 

## Figures and Tables

**Table 1:** Water contact angle (WCA) values of all investigated samples.

	Neat	CA	HNT
**CS**	85°	78°	81°
**PC**	45°	49°	58°
**CS:PC**	64°	79°	84°

**Table 2:** Calculated values of linear attenuation coefficient *K_750_* (mm^−1^) for all investigated samples.

	Neat	CA	HNT
CS	0.327	0.309	0.774
PC	0.330	0.513	4.513
CS:PC	0.395	0.396	1.288

**Table 3:** Assignment of main IR absorption bands of Chitosan [14] and Pectin [34].

Chitosan		Pectin	
ν cm^−1^	Attribution	ν cm^−1^	Attribution
2927	symmetric C-H stretching	2929	symmetric-CH_2_ stretching
1736	>C=O stretching	1739	-O-C=O
1626	C=O of ion COO stretching of secondary amide group (amide I)	1634	-O^(−)^-C=O
1530	N-H bending (residue of amide II)	1015	-C-O-C-
1395	C=N stretching (amide III band)	955	rhamnogalacturonan (uronic acid)
1353	N-H in plan deformation	923	d-glucopyranosyl
955	piranose ring	890, 852	α-, β-glucosidic linkage
890	C-N fingerprint band	832	a-D- mannopyranose

## Data Availability

Data sharing not applicable.

## Sample Availability

Samples of the compounds CS, PC, CS:PC, with and without CA and HNT, are available from the authors.

## References

[1] European Commission (2019). The European Green Deal COM(2019) 640 Final.

[2] Scott G., Gilead D. (1995). Degradable Polymers.

[3] Gross R.A., Kalra B. (2002). Biodegradable Polymers for the Environment. Science.

[4] Bastioli C. (2005). Handbook of Biodegradable Polymers.

[5] Ebnesajjad S. (2012). Handbook of Biopolymers and Biodegradable Plastics.

[6] Martau G.A., Mihai M., Vodnar D.C. (2019). The use of chitosan, alginate, and pectin in the biomedical and food sector—biocompatibility, bioadhesiveness and biodegradability. Polymers.

[7] Elsabee M.Z., Abdou E.S. (2013). Chitosan based edible films and coatings: A review. Mater. Sci. Eng. C.

[8] Souza H.K.S., Campiña J.M., Sousa A.M.M., Silva F., Gonçalves M.P. (2013). Ultrasound assisted preparation of size-controlled chitosan nanoparticles: Characterization and fabrication of transparent biofilms. Food Hydrocoll..

[9] Guo Z., Xing R., Liu S., Zhong Z., Ji X., Wang L., Li P. (2008). The influence of molecular weight of quaternized chitosan on antifungal activity. Carbohyd. Polym..

[10] Arancibia M.Y., López-Caballero M.E., Gómez-Guillén M.C., Fernández-García M., Fernández-Martín F., Montero P. (2015). Antimicrobial and rheological properties of chitosan as affected by extracting conditions and humidity exposure. LWT-Food Sci. Technol..

[11] Águila-Almanza E., Sze ShinLow H., Hernández-Cocoletzi A., Atonal-Sandoval E., Rubio-Rosas J., Violante-González J., Show P.L. (2021). Facile and green approach in managing sand crab carapace biowaste for obtention of high deacetylation percentage chitosan. J. Environ. Chem. Eng..

[12] Sun L., Sun J., Chen L., Niu P., Yang X., Guo Y. (2017). Preparation and characterization of chitosan film incorporated with thinned young apple polyphenols as an active packaging material. Carbohyd. Polym..

[13] Branca C., D’Angelo G., Crupi C., Khouzami K., Rifici S., Ruello G., Wanderlingh U. (2016). Role of the OH and NH vibrational groups in polysaccharide-nanocomposite interactions: A FTIR-ATR study on chitosan andchitosan/clayfilms. Polymer.

[14] Wu H., Lei Y., Lu J., Zhu R., Xiao D., Jiao C., Xia R., Zhang Z., Shen G., Liu Y. (2019). Effect of citric acid induced crosslinking on the structure and properties of potato starch/chitosan composite films. Food Hydrocoll..

[15] Priyadarshi R., Sauraj, Kumar B., Negi Y.S. (2018). Chitosan film incorporated with citric acid and glycerol as an active packaging material for extension of green chilli shelf life. Carbohyd. Polym..

[16] Yapo B.M. (2011). Pectin substances: From simple pectic polysaccharides to complex pectins-a new hypothetical model. Carbohyd. Polym..

[17] Lara-Espinoza C., Carvajal-Millán E., Balandrán-Quintana R., López-Franco Y., Rascón-Chu A. (2018). Pectin and Pectin-Based Composite Materials: Beyond Food Texture. Molecules.

[18] Fraeye I., Doungla E., Duvetter T., Moldenaers P., Loey A.V., Hendrickx M. (2009). Influence of Intrinsic and Extrinsic Factors on Rheology of Pectin–calcium Gels. Food Hydrocoll..

[19] Infurna G., Cavallaro G., Lazzara G., Milioto S., Dintcheva N.T. (2020). Bionanocomposite films containing halloysite nanotubes and natural antioxidants with enhanced performance and durability as promising materials for cultural heritage protection. Polymers.

[20] Chao C., Guan H., Zhang J., Liu Y., Zhao Y., Zhang B. (2018). Immobilization of laccase onto porous polyvinyl alcohol/halloysite hybrid beads for dye removal. Water Sci. Technol..

[21] Cavallaro G., Micciulla S., Chiappisi L., Lazzara G. (2021). Chitosan-based smart hybrid materials: A physico-chemical perspective. J. Mater. Chem. B.

[22] Zhao X., Zhou C., Liu M. (2020). Self-assembled structures of halloysite nanotubes: Towards the development of high-performance biomedical materials. J. Mater. Chem. B.

[23] Liu M., Wu C., Jiao Y., Xiong S., Zhou C. (2013). Chitosan-Halloysite Nanotubes Nanocomposite Scaffolds for Tissue Engineering. J. Mater. Chem. B.

[24] Lisuzzo L., Caruso M.R., Cavallaro G., Milioto S., Lazzara G. (2021). Hydroxypropyl cellulose films filled with halloysite nanotubes/wax hybrid microspheres. Ind. Eng. Chem. Res..

[25] Lisuzzo L., Hueckel T., Cavallaro G., Sacanna S., Lazzara G. (2021). Pickering emulsions based on wax and halloysite nanotubes: An ecofriendly protocol for the treatment of archeological woods. ACS Appl. Mater. Interfaces.

[26] Cavallaro G., Milioto S., Lazzara G. (2020). Halloysite nanotubes: Interfacial properties and applications in cultural heritage. Langmuir.

[27] Lisuzzo L., Cavallaro G., Milioto S., Lazzara G. (2020). Effects of halloysite content on the thermo-mechanical performances of composite bioplastics. Appl. Clay Sci..

[28] Naumenko E.A., Guryanov I.D., Yendluri R., Lvov Y.M., Fakhrullin R.F. (2016). Clay nanotube-biopolymer composite scaffolds for tissue engineering. Nanoscale.

[29] Lvov Y., Panchal A., Fu Y., Fakhrullin R., Kryuchkova M., Batasheva S., Stavitskaya A., Glotov A., Vinokurov V. (2019). Interfacial self-assembly in halloysite nanotube composites. Langmuir.

[30] Cavallaro G., Danilushkina A.A., Evtugyn V.G., Lazzara G., Milioto S., Parisi F., Rozhina E.V., Fakhrullin R.F. (2017). Halloysite nanotubes: Controlled access and release by smart gates. Nanomaterials.

[31] Rozhina E., Batasheva S., Miftakhova R., Yan X., Vikulina A., Volodkin D., Fakhrullin R. (2021). Comparative cytotoxicity of kaolinite, halloysite, multiwalled carbon nanotubes and graphene oxide. Appl. Clay Sci..

[32] Rozhina E., Panchal A., Akhatova F., Lvov Y., Fakhrullin R. (2020). Cytocompatibility and cellular uptake of alkylsilane-modified hydrophobic halloysite nanotubes. Appl. Clay Sci..

[33] Lisuzzo L., Cavallaro G., Milioto S., Lazzara G. (2019). Layered composite based on halloysite and natural polymers: A carrier for the PH controlled release of drugs. New J. Chem..

[34] Younis H.G.R., Zhao G. (2019). Physicochemical properties of the edible films from the blends of high methoxyl apple pectin and chitosan. Int. J. Biol. Macromol..

[35] Szyma’ nska E.K., Winnicka K. (2015). Stability of chitosan-a challenge for pharmaceutical and biomedical applications. Mar. Drugs.

[36] Einhorn-Stoll U., Kastner H., Urbisch A., Kroh L.W., Drusch S. (2019). Thermal degradation of citrus pectin in low-moisture environment—Influence of acidic and alkaline pre-treatment. Food Hydrocoll..

[37] Cavallaro G., Lazzara G., Milioto S. (2011). Dispersions of nanoclays of di_erent shapes into aqueous and solid biopolymeric matrices. Extended physicochemical study. Langmuir.

[38] Pérez Amaro L., Cicogna F., Passaglia E., Morici E., Oberhauser W., Al Malaika S., Dintcheva N.T., Coiai S. (2016). Thermo-oxidative stabilization of poly(lactic acid) with antioxidant intercalated layered double hydroxides. Polym. Degrad. Stab..

[39] Dintcheva N.T., Al-Malaika S. (2020). Photo-stabilization of biopolymers-based nanocomposites with UV-modified layered silicates. Polym. Degrad. Stab..

[40] Dintcheva N.T., D’Anna F. (2019). Anti-/Pro- oxidant behaviour of naturally occurring molecules in polymers and biopolymers: A brief review. ACS Sustain. Chem. Eng..

[41] Infurna G., Cavallaro G., Lazzara G., Milioto S., Dintcheva N.T. (2021). Taking advantages of different processing techniques and crosslinking agent on the chitosan film performance. Int. J. Biol. Macromol..

